# 1-[5-(4-Bromo­phen­yl)-3-(4-fluoro­phen­yl)-4,5-dihydro-1*H*-pyrazol-1-yl]butan-1-one

**DOI:** 10.1107/S1600536812034368

**Published:** 2012-08-08

**Authors:** Hoong-Kun Fun, Wan-Sin Loh, M. Sapnakumari, B. Narayana, B. K. Sarojini

**Affiliations:** a X-ray Crystallography Unit, School of Physics, Universiti Sains Malaysia, 11800 USM, Penang, Malaysia; b Department of Studies in Chemistry, Mangalore University, Mangalagangotri 574 199, India; c Department of Chemistry, P.A. College of Engineering, Nadupadavu, Mangalore 574 153, India

## Abstract

In the title compound, C_19_H_18_BrFN_2_O, the benzene rings form dihedral angles of 5.38 (7) and 85.48 (7)° with the mean plane of the 4,5-dihydro-1*H*-pyrazole ring (r.m.s. deviation = 0.0849 Å), which approximates to an envelope conformation with the –CH_2_– group as the flap. The dihedral angle between the benzene rings is 82.86 (7)°. In the crystal, C—H⋯F and C—H⋯O hydrogen bonds link the mol­ecules to form inversion dimers and together these generate chains along [011]. The crystal packing also features C—H⋯π inter­actions.

## Related literature

For background to pyrazoline derivatives, see: Fun *et al.* (2010 ▶); Samshuddin *et al.* (2011 ▶). For related structures, see: Fun, Quah *et al.* (2012 ▶); Fun, Loh *et al.* (2012 ▶). For the stability of the temperature controller used for the data collection, see: Cosier & Glazer (1986 ▶).
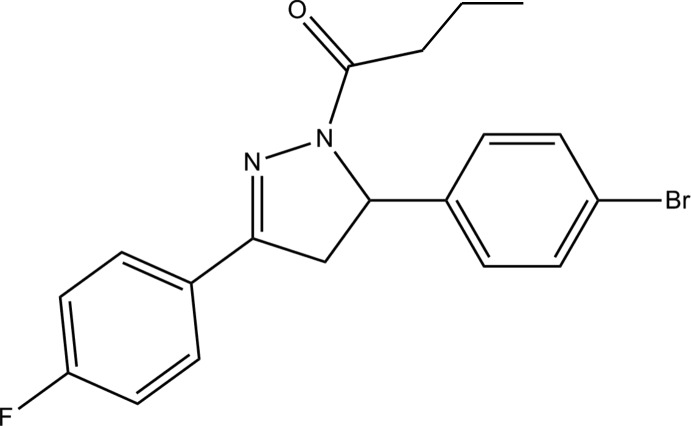


## Experimental

### 

#### Crystal data

C_19_H_18_BrFN_2_O
*M* *_r_* = 389.26
Triclinic, 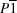

*a* = 6.7502 (3) Å
*b* = 10.1253 (5) Å
*c* = 13.7792 (8) Å
α = 105.354 (1)°
β = 98.976 (1)°
γ = 107.369 (1)°
*V* = 838.01 (7) Å^3^
*Z* = 2
Mo *K*α radiation
μ = 2.47 mm^−1^
*T* = 100 K
0.28 × 0.23 × 0.08 mm

#### Data collection

Bruker SMART APEXII DUO CCD diffractometer
Absorption correction: multi-scan (*SADABS*; Bruker, 2009 ▶) *T*_min_ = 0.546, *T*_max_ = 0.823
17838 measured reflections
4816 independent reflections
4458 reflections with *I* > 2σ(*I*)
*R*_int_ = 0.023

#### Refinement

*R*[*F* ^2^ > 2σ(*F* ^2^)] = 0.025
*wR*(*F* ^2^) = 0.066
*S* = 1.06
4816 reflections
218 parameters
H-atom parameters constrained
Δρ_max_ = 0.49 e Å^−3^
Δρ_min_ = −0.63 e Å^−3^

### 

Data collection: *APEX2* (Bruker, 2009 ▶); cell refinement: *SAINT* (Bruker, 2009 ▶); data reduction: *SAINT*; program(s) used to solve structure: *SHELXTL* (Sheldrick, 2008 ▶); program(s) used to refine structure: *SHELXTL*; molecular graphics: *SHELXTL*; software used to prepare material for publication: *SHELXTL* and *PLATON* (Spek, 2009 ▶).

## Supplementary Material

Crystal structure: contains datablock(s) global, I. DOI: 10.1107/S1600536812034368/hb6918sup1.cif

Structure factors: contains datablock(s) I. DOI: 10.1107/S1600536812034368/hb6918Isup2.hkl

Supplementary material file. DOI: 10.1107/S1600536812034368/hb6918Isup3.cml

Additional supplementary materials:  crystallographic information; 3D view; checkCIF report

## Figures and Tables

**Table 1:** Hydrogen-bond geometry (Å, °) *Cg*1 and *Cg*2 are the centroids of the C1–C5 and C10–C15 benzene rings, respectively.

*D*—H⋯*A*	*D*—H	H⋯*A*	*D*⋯*A*	*D*—H⋯*A*
C11—H11*A*⋯F1^i^	0.95	2.37	3.1873 (17)	144
C14—H14*A*⋯O1^ii^	0.95	2.57	3.1832 (16)	122
C5—H5*A*⋯*Cg*2^iii^	0.95	2.68	3.5453 (15)	152
C17—H17*B*⋯*Cg*1^iv^	0.99	2.70	3.5488 (14)	144

Symmetry codes: (i) 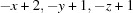; (ii) 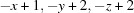; (iii) 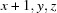; (iv) 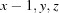.

**Fig. 1.**
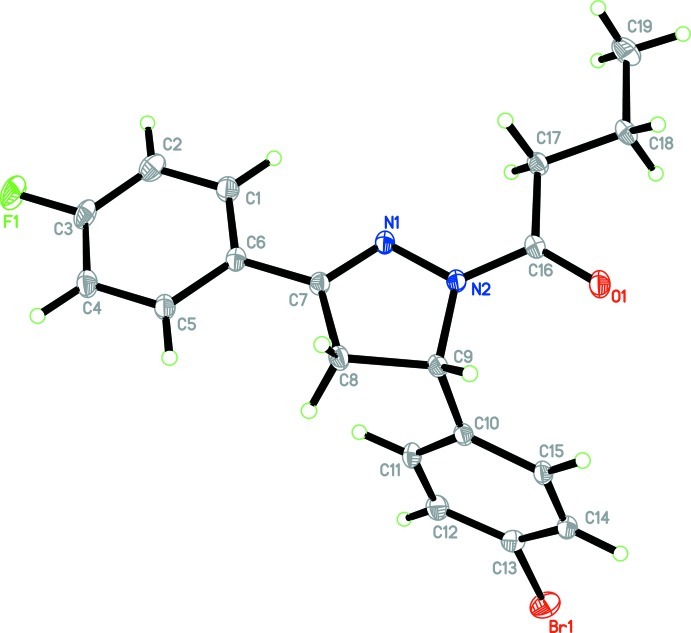
The molecular structure of the title compound, showing 50% probability displacement ellipsoids.

**Fig. 2.**
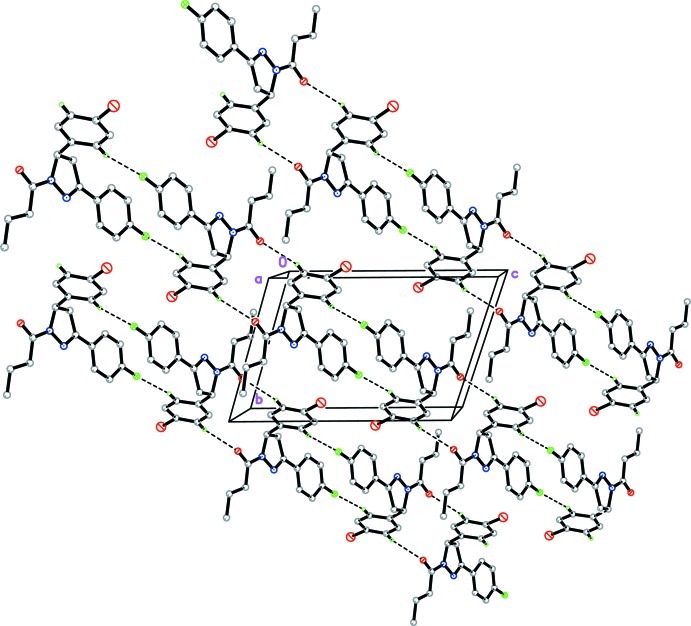
The crystal packing of the title compound, viewed along the a axis. H atoms not involved in the intermolecular interactions (dashed lines) have been omitted for clarity.

## supplementary crystallographic information

### Comment

In continuation of our work on synthesis of pyrazoline derivatives (Fun *et al.*, 2010; Samshuddin *et al.*, 2011), the title compound is prepared and its crystal structure is reported.

In the title compound, Fig. 1, the benzene rings (C1–C6 & C10–C15) form dihedral angles of 5.38 (7) and 85.48 (7)°, respectively, with the mean plane of 4,5-dihydro-1*H*-pyrazole ring (N1/N2/C7–C9, r.m.s. deviation = 0.0849 Å). The dihedral angle between the two benzene rings is 82.86 (7) °. Bond lengths and angles are comparable with those in related structures (Fun, Quah *et al.*, 2012; Fun, Loh *et al.*, 2012).

In the crystal packing as shown in Fig. 2, C11—H11A···F1 and C14—H14A···O1 hydrogen bonds (Table 1) link the molecules to form dimers, generating chains along the [011]. The crystal packing is further consolidated by C17—H17B···*Cg*1 and C5—H5A···*Cg*2 (Table 1) interactions, where *Cg*1 and *Cg*2 are the centroids of C1–C5 and C10–C15 benzene rings, respectively.

### Experimental

A mixture of (2*E*)-3-(4-bromophenyl)-1-(4-fluorophenyl)prop-2-en-1-one (3.05 g, 0.01 mol) and hydrazine hydrate (0.48 ml, 0.01 mol) in 30 ml butyric acid was refluxed for 6 h. The reaction mixture was cooled and poured into 50 ml ice-cold water. The precipitate was collected by filtration and purified by recrystallization from ethanol. Colourless plates were grown from acetone solution by slow evaporation method. *M. p.*: 383–385 K.

### Refinement

All the H atoms were located geometrically and were refined using a riding model with *U*_iso_(H) = 1.2 or 1.5 *U*_eq_(C) [C–H = 0.95 to 0.99 Å]. A rotating group model was applied to the methyl group. In the final refinement, eighteen outliners were omitted, 3 - 3 7, 0 - 3 10, 4 0 3, 2 - 4 9, 2 0 2, 4 - 1 4, 1 0 0, -2 4 6, 2 5 1, -1 2 4, 1 0 5, -2 2 6, -2 5 4, 0 6 3, -1 - 1 8, 1 - 3 9, 15 2 and 1 2 1.

### Crystal data

**Table:**

C_19_H_18_BrFN_2_O	*Z* = 2
*M**_r_* = 389.26	*F*(000) = 396
Triclinic, *P*1	*D*_x_ = 1.543 Mg m^−^^3^
Hall symbol: -P 1	Mo *K*α radiation, λ = 0.71073 Å
*a* = 6.7502 (3) Å	Cell parameters from 9994 reflections
*b* = 10.1253 (5) Å	θ = 3.1–33.0°
*c* = 13.7792 (8) Å	µ = 2.47 mm^−^^1^
α = 105.354 (1)°	*T* = 100 K
β = 98.976 (1)°	Plate, colourless
γ = 107.369 (1)°	0.28 × 0.23 × 0.08 mm
*V* = 838.01 (7) Å^3^	

### Data collection

**Table:**

Bruker SMART APEXII DUO CCD diffractometer	4816 independent reflections
Radiation source: fine-focus sealed tube	4458 reflections with *I* > 2σ(*I*)
Graphite monochromator	*R*_int_ = 0.023
φ and ω scans	θ_max_ = 30.0°, θ_min_ = 2.2°
Absorption correction: multi-scan (*SADABS*; Bruker, 2009)	*h* = −9→9
*T*_min_ = 0.546, *T*_max_ = 0.823	*k* = −14→14
17838 measured reflections	*l* = −19→19

### Refinement

**Table:**

Refinement on *F*^2^	Primary atom site location: structure-invariant direct methods
Least-squares matrix: full	Secondary atom site location: difference Fourier map
*R*[*F*^2^ > 2σ(*F*^2^)] = 0.025	Hydrogen site location: inferred from neighbouring sites
*wR*(*F*^2^) = 0.066	H-atom parameters constrained
*S* = 1.06	*w* = 1/[σ^2^(*F*_o_^2^) + (0.0342*P*)^2^ + 0.3618*P*] where *P* = (*F*_o_^2^ + 2*F*_c_^2^)/3
4816 reflections	(Δ/σ)_max_ = 0.001
218 parameters	Δρ_max_ = 0.49 e Å^−^^3^
0 restraints	Δρ_min_ = −0.63 e Å^−^^3^

### Special details

**Table:**

Experimental. The crystal was placed in the cold stream of an Oxford Cryosystems Cobra open-flow nitrogen cryostat (Cosier & Glazer, 1986) operating at 100.0 (1) K.
Geometry. All e.s.d.'s (except the e.s.d. in the dihedral angle between two l.s. planes) are estimated using the full covariance matrix. The cell e.s.d.'s are taken into account individually in the estimation of e.s.d.'s in distances, angles and torsion angles; correlations between e.s.d.'s in cell parameters are only used when they are defined by crystal symmetry. An approximate (isotropic) treatment of cell e.s.d.'s is used for estimating e.s.d.'s involving l.s. planes.
Refinement. Refinement of *F*^2^ against ALL reflections. The weighted *R*-factor *wR* and goodness of fit *S* are based on *F*^2^, conventional *R*-factors *R* are based on *F*, with *F* set to zero for negative *F*^2^. The threshold expression of *F*^2^ > σ(*F*^2^) is used only for calculating *R*-factors(gt) *etc*. and is not relevant to the choice of reflections for refinement. *R*-factors based on *F*^2^ are statistically about twice as large as those based on *F*, and *R*- factors based on ALL data will be even larger.

### Fractional atomic coordinates and isotropic or equivalent isotropic displacement parameters (Å^2^)

**Table:**

	*x*	*y*	*z*	*U*_iso_*/*U*_eq_	
Br1	0.29346 (2)	1.105034 (15)	0.629792 (11)	0.02435 (5)	
F1	1.22640 (16)	0.30984 (10)	0.49188 (7)	0.02653 (19)	
O1	0.44837 (15)	0.73712 (10)	0.95847 (7)	0.01715 (18)	
N1	0.69619 (17)	0.56573 (11)	0.78382 (8)	0.01292 (18)	
N2	0.65164 (17)	0.67234 (11)	0.85431 (8)	0.01310 (19)	
C1	0.8653 (2)	0.39253 (14)	0.64641 (10)	0.0171 (2)	
H1A	0.7351	0.3438	0.6608	0.020*	
C2	0.9522 (2)	0.31297 (15)	0.57824 (11)	0.0201 (2)	
H2A	0.8834	0.2100	0.5457	0.024*	
C3	1.1414 (2)	0.38745 (16)	0.55897 (10)	0.0184 (2)	
C4	1.2486 (2)	0.53618 (15)	0.60412 (10)	0.0175 (2)	
H4A	1.3788	0.5836	0.5892	0.021*	
C5	1.1603 (2)	0.61528 (14)	0.67260 (10)	0.0150 (2)	
H5A	1.2310	0.7182	0.7049	0.018*	
C6	0.96862 (19)	0.54438 (13)	0.69403 (9)	0.0133 (2)	
C7	0.87764 (19)	0.62814 (13)	0.76656 (9)	0.0127 (2)	
C8	0.98525 (19)	0.78883 (13)	0.83134 (10)	0.0149 (2)	
H8A	1.0376	0.8493	0.7883	0.018*	
H8B	1.1065	0.8046	0.8891	0.018*	
C9	0.80040 (19)	0.82300 (13)	0.87232 (9)	0.0131 (2)	
H9A	0.8511	0.8801	0.9484	0.016*	
C10	0.68978 (19)	0.89988 (13)	0.81387 (9)	0.0129 (2)	
C11	0.6768 (2)	0.87756 (14)	0.70818 (10)	0.0165 (2)	
H11A	0.7482	0.8192	0.6735	0.020*	
C12	0.5613 (2)	0.93907 (14)	0.65294 (10)	0.0183 (2)	
H12A	0.5527	0.9229	0.5810	0.022*	
C13	0.4586 (2)	1.02469 (14)	0.70504 (10)	0.0167 (2)	
C14	0.4748 (2)	1.05360 (13)	0.81074 (10)	0.0158 (2)	
H14A	0.4084	1.1155	0.8458	0.019*	
C15	0.59010 (19)	0.99012 (13)	0.86442 (9)	0.0143 (2)	
H15A	0.6012	1.0085	0.9367	0.017*	
C16	0.48284 (19)	0.63837 (13)	0.89729 (9)	0.0128 (2)	
C17	0.34922 (19)	0.47749 (13)	0.86741 (9)	0.0137 (2)	
H17A	0.4423	0.4249	0.8877	0.016*	
H17B	0.2912	0.4363	0.7909	0.016*	
C18	0.1640 (2)	0.45162 (14)	0.91894 (10)	0.0157 (2)	
H18A	0.0674	0.5007	0.8965	0.019*	
H18B	0.2213	0.4959	0.9954	0.019*	
C19	0.0363 (2)	0.28890 (16)	0.89136 (11)	0.0226 (3)	
H19A	−0.0778	0.2765	0.9279	0.034*	
H19B	0.1321	0.2396	0.9123	0.034*	
H19C	−0.0274	0.2459	0.8161	0.034*	

### Atomic displacement parameters (Å^2^)

**Table:**

	*U*^11^	*U*^22^	*U*^33^	*U*^12^	*U*^13^	*U*^23^
Br1	0.02830 (8)	0.02231 (8)	0.02494 (8)	0.01276 (6)	0.00379 (5)	0.00926 (5)
F1	0.0359 (5)	0.0316 (5)	0.0200 (4)	0.0222 (4)	0.0148 (4)	0.0051 (3)
O1	0.0176 (4)	0.0163 (4)	0.0177 (4)	0.0058 (3)	0.0095 (3)	0.0033 (3)
N1	0.0128 (4)	0.0133 (4)	0.0136 (4)	0.0056 (4)	0.0056 (4)	0.0037 (4)
N2	0.0127 (4)	0.0109 (4)	0.0156 (5)	0.0035 (4)	0.0068 (4)	0.0030 (4)
C1	0.0156 (5)	0.0162 (5)	0.0185 (6)	0.0053 (4)	0.0053 (5)	0.0042 (5)
C2	0.0235 (6)	0.0180 (6)	0.0174 (6)	0.0097 (5)	0.0041 (5)	0.0018 (5)
C3	0.0242 (6)	0.0247 (6)	0.0122 (5)	0.0163 (5)	0.0071 (5)	0.0054 (5)
C4	0.0169 (6)	0.0244 (6)	0.0167 (5)	0.0108 (5)	0.0085 (5)	0.0092 (5)
C5	0.0142 (5)	0.0174 (5)	0.0153 (5)	0.0070 (4)	0.0058 (4)	0.0058 (4)
C6	0.0128 (5)	0.0162 (5)	0.0123 (5)	0.0069 (4)	0.0041 (4)	0.0047 (4)
C7	0.0110 (5)	0.0134 (5)	0.0137 (5)	0.0047 (4)	0.0036 (4)	0.0040 (4)
C8	0.0110 (5)	0.0142 (5)	0.0187 (5)	0.0032 (4)	0.0066 (4)	0.0037 (4)
C9	0.0117 (5)	0.0120 (5)	0.0147 (5)	0.0026 (4)	0.0059 (4)	0.0032 (4)
C10	0.0126 (5)	0.0115 (5)	0.0134 (5)	0.0027 (4)	0.0057 (4)	0.0027 (4)
C11	0.0190 (6)	0.0170 (5)	0.0154 (5)	0.0077 (5)	0.0096 (5)	0.0041 (4)
C12	0.0224 (6)	0.0186 (6)	0.0151 (5)	0.0072 (5)	0.0083 (5)	0.0054 (5)
C13	0.0168 (5)	0.0142 (5)	0.0194 (6)	0.0050 (4)	0.0054 (5)	0.0063 (4)
C14	0.0151 (5)	0.0131 (5)	0.0193 (6)	0.0046 (4)	0.0078 (4)	0.0036 (4)
C15	0.0145 (5)	0.0132 (5)	0.0142 (5)	0.0034 (4)	0.0072 (4)	0.0026 (4)
C16	0.0113 (5)	0.0154 (5)	0.0124 (5)	0.0046 (4)	0.0041 (4)	0.0052 (4)
C17	0.0123 (5)	0.0140 (5)	0.0146 (5)	0.0035 (4)	0.0048 (4)	0.0048 (4)
C18	0.0127 (5)	0.0191 (5)	0.0165 (5)	0.0039 (4)	0.0063 (4)	0.0080 (4)
C19	0.0180 (6)	0.0216 (6)	0.0240 (7)	−0.0006 (5)	0.0067 (5)	0.0087 (5)

### Geometric parameters (Å, º)

**Table:**

Br1—C13	1.8991 (13)	C9—C10	1.5177 (17)
F1—C3	1.3606 (14)	C9—H9A	1.0000
O1—C16	1.2322 (15)	C10—C15	1.3960 (16)
N1—C7	1.2901 (16)	C10—C11	1.3978 (17)
N1—N2	1.3888 (13)	C11—C12	1.3894 (19)
N2—C16	1.3608 (15)	C11—H11A	0.9500
N2—C9	1.4846 (15)	C12—C13	1.3902 (17)
C1—C2	1.3891 (17)	C12—H12A	0.9500
C1—C6	1.4013 (17)	C13—C14	1.3872 (18)
C1—H1A	0.9500	C14—C15	1.3918 (18)
C2—C3	1.381 (2)	C14—H14A	0.9500
C2—H2A	0.9500	C15—H15A	0.9500
C3—C4	1.375 (2)	C16—C17	1.5127 (17)
C4—C5	1.3957 (16)	C17—C18	1.5219 (16)
C4—H4A	0.9500	C17—H17A	0.9900
C5—C6	1.3975 (17)	C17—H17B	0.9900
C5—H5A	0.9500	C18—C19	1.5232 (19)
C6—C7	1.4660 (16)	C18—H18A	0.9900
C7—C8	1.5148 (17)	C18—H18B	0.9900
C8—C9	1.5394 (16)	C19—H19A	0.9800
C8—H8A	0.9900	C19—H19B	0.9800
C8—H8B	0.9900	C19—H19C	0.9800
			
C7—N1—N2	107.76 (10)	C15—C10—C9	120.04 (11)
C16—N2—N1	122.03 (10)	C11—C10—C9	121.35 (10)
C16—N2—C9	125.22 (10)	C12—C11—C10	121.24 (11)
N1—N2—C9	112.69 (9)	C12—C11—H11A	119.4
C2—C1—C6	120.32 (12)	C10—C11—H11A	119.4
C2—C1—H1A	119.8	C11—C12—C13	118.64 (12)
C6—C1—H1A	119.8	C11—C12—H12A	120.7
C3—C2—C1	118.28 (12)	C13—C12—H12A	120.7
C3—C2—H2A	120.9	C14—C13—C12	121.63 (12)
C1—C2—H2A	120.9	C14—C13—Br1	119.20 (9)
F1—C3—C4	118.22 (12)	C12—C13—Br1	119.17 (10)
F1—C3—C2	118.38 (12)	C13—C14—C15	118.73 (11)
C4—C3—C2	123.40 (12)	C13—C14—H14A	120.6
C3—C4—C5	117.96 (12)	C15—C14—H14A	120.6
C3—C4—H4A	121.0	C14—C15—C10	121.14 (11)
C5—C4—H4A	121.0	C14—C15—H15A	119.4
C4—C5—C6	120.54 (12)	C10—C15—H15A	119.4
C4—C5—H5A	119.7	O1—C16—N2	119.59 (11)
C6—C5—H5A	119.7	O1—C16—C17	123.68 (11)
C5—C6—C1	119.50 (11)	N2—C16—C17	116.72 (10)
C5—C6—C7	120.20 (11)	C16—C17—C18	112.50 (10)
C1—C6—C7	120.30 (11)	C16—C17—H17A	109.1
N1—C7—C6	121.06 (11)	C18—C17—H17A	109.1
N1—C7—C8	113.62 (10)	C16—C17—H17B	109.1
C6—C7—C8	125.25 (10)	C18—C17—H17B	109.1
C7—C8—C9	101.79 (9)	H17A—C17—H17B	107.8
C7—C8—H8A	111.4	C17—C18—C19	111.82 (11)
C9—C8—H8A	111.4	C17—C18—H18A	109.3
C7—C8—H8B	111.4	C19—C18—H18A	109.3
C9—C8—H8B	111.4	C17—C18—H18B	109.3
H8A—C8—H8B	109.3	C19—C18—H18B	109.3
N2—C9—C10	110.08 (10)	H18A—C18—H18B	107.9
N2—C9—C8	100.37 (9)	C18—C19—H19A	109.5
C10—C9—C8	114.80 (10)	C18—C19—H19B	109.5
N2—C9—H9A	110.4	H19A—C19—H19B	109.5
C10—C9—H9A	110.4	C18—C19—H19C	109.5
C8—C9—H9A	110.4	H19A—C19—H19C	109.5
C15—C10—C11	118.55 (11)	H19B—C19—H19C	109.5
			
C7—N1—N2—C16	−172.51 (11)	C7—C8—C9—N2	17.81 (11)
C7—N1—N2—C9	10.01 (13)	C7—C8—C9—C10	−100.16 (11)
C6—C1—C2—C3	−0.1 (2)	N2—C9—C10—C15	94.86 (13)
C1—C2—C3—F1	−179.72 (12)	C8—C9—C10—C15	−152.80 (11)
C1—C2—C3—C4	0.3 (2)	N2—C9—C10—C11	−82.31 (14)
F1—C3—C4—C5	179.79 (11)	C8—C9—C10—C11	30.03 (16)
C2—C3—C4—C5	−0.2 (2)	C15—C10—C11—C12	−2.23 (19)
C3—C4—C5—C6	−0.02 (19)	C9—C10—C11—C12	174.98 (12)
C4—C5—C6—C1	0.20 (19)	C10—C11—C12—C13	0.3 (2)
C4—C5—C6—C7	179.61 (11)	C11—C12—C13—C14	2.1 (2)
C2—C1—C6—C5	−0.13 (19)	C11—C12—C13—Br1	−178.25 (10)
C2—C1—C6—C7	−179.54 (12)	C12—C13—C14—C15	−2.6 (2)
N2—N1—C7—C6	−179.32 (10)	Br1—C13—C14—C15	177.84 (9)
N2—N1—C7—C8	3.50 (14)	C13—C14—C15—C10	0.53 (19)
C5—C6—C7—N1	176.94 (12)	C11—C10—C15—C14	1.81 (18)
C1—C6—C7—N1	−3.66 (18)	C9—C10—C15—C14	−175.44 (11)
C5—C6—C7—C8	−6.23 (18)	N1—N2—C16—O1	−179.08 (11)
C1—C6—C7—C8	173.18 (12)	C9—N2—C16—O1	−1.92 (18)
N1—C7—C8—C9	−14.48 (14)	N1—N2—C16—C17	2.02 (16)
C6—C7—C8—C9	168.48 (11)	C9—N2—C16—C17	179.18 (11)
C16—N2—C9—C10	−74.12 (14)	O1—C16—C17—C18	1.59 (17)
N1—N2—C9—C10	103.27 (11)	N2—C16—C17—C18	−179.55 (10)
C16—N2—C9—C8	164.49 (11)	C16—C17—C18—C19	−177.81 (11)
N1—N2—C9—C8	−18.12 (12)		

### Hydrogen-bond geometry (Å, º)

**Table:**

*D*—H···*A*	*D*—H	H···*A*	*D*···*A*	*D*—H···*A*
C11—H11*A*···F1^i^	0.95	2.37	3.1873 (17)	144
C14—H14*A*···O1^ii^	0.95	2.57	3.1832 (16)	122
C5—H5*A*···*Cg*2^iii^	0.95	2.68	3.5453 (15)	152
C17—H17*B*···*Cg*1^iv^	0.99	2.70	3.5488 (14)	144

Symmetry codes: (i) −*x*+2, −*y*+1, −*z*+1; (ii) −*x*+1, −*y*+2, −*z*+2; (iii) *x*+1, *y*, *z*; (iv) *x*−1, *y*, *z*.
